# Primary hepatic lymphoma presenting as fulminant hepatic failure with hyperferritinemia: A case report

**DOI:** 10.1186/1752-1947-2-279

**Published:** 2008-08-19

**Authors:** Fyeza S Haider, Robert Smith, Sharif Khan

**Affiliations:** 1 Department of Internal Medicine/Hospitalist Medicine, Geisinger Medical Center, Danville, PA 17821, USA; 2 Department of Gastroenterology and Hepatology, Geisinger Medical Center, Danville, PA 17821, USA; 3 Department of Hematology and Oncology, Geisinger Medical Center, Danville, PA 17821, USA

## Abstract

**Introduction:**

Primary hepatic lymphoma is an unusual form of non-Hodgkin's lymphoma that usually presents with constitutional symptoms, hepatomegaly and signs of cholestatic jaundice. Diffuse hepatic infiltration is uncommon and presentation with acute hepatic failure even more rare. The presence of markedly elevated ferritin levels can complicate the evaluation process and suggest alternative diagnoses.

We present the case of a middle-aged woman exhibiting pancytopenia, hyperferritinemia and rapidly deteriorating to develop acute hepatic failure. Her initial clinical picture led to a working diagnosis of adult onset Still's disease with probable hemophagocytic syndrome before her worsening liver function necessitated a percutaneous liver biopsy and establishment of the final diagnosis of primary hepatic lymphoma.

**Conclusion:**

Primary hepatic lymphoma is an uncommon malignancy and its manifestation as progressive hepatitis or acute fulminant hepatic failure can be difficult to diagnose. The presence of constitutional symptoms, pancytopenia and high ferritin levels can complicate the evaluation process. A liver biopsy early in the course of liver dysfunction may establish the diagnosis without a higher risk of bleeding complications seen once liver failure sets in.

## Introduction

Primary Hepatic Lymphoma (PHL) is a rare variant of non-Hodgkin's lymphoma with an incidence of < 1%. The presence of diffuse hepatic involvement is uncommon and therefore presentation as hepatocellular jaundice or acute fulminant hepatic failure is rare.

We present a case where persistent fever, non-specific symptoms, pancytopenia and strikingly high levels of serum ferritin preceded the presentation of acute liver failure. Due to these findings, alternative diagnoses were entertained including hemophagocytic syndrome in association with adult onset Still's disease (AOSD).

## Case presentation

A 53-year-old Caucasian woman was transferred to our facility for 3 weeks of intermittent fevers, chills, weight loss, myalgias and arthralgias. She had mild epigastric discomfort with nausea and vomiting. Dalteparin and warfarin were started for a recently diagnosed pulmonary embolism. Her past history was remarkable for diabetes mellitus type 2, hypertension and villous adenoma of the colon. Subsequent colonoscopies were normal. Except for mild epigastric tenderness, her physical examination findings were normal. White blood cell (WBC) count was 4.5 K/μl, hemoglobin 10 g/dl, hematocrit 28.6% and platelets had fallen to 95 K/μl from a baseline of 129 K/μl. Aspartate aminotransferase (AST) was 67 U/liter and albumin 2.9 g/liter. Repeat imaging did not show a pulmonary embolism but her spleen was enlarged (16.5 cm) with a focal infarct. Argatroban, originally started for possible heparin induced thrombocytopenia, was discontinued when venous Dopplers, Ventilation-perfusion scan and heparin antibodies were normal. Transesophageal echocardiogram and all cultures were negative.

She continued to have fever up to 39.5°C. Lactate dehydrogenase (LDH) was 2075 U/liter and serum ferritin was 87,207 ng/ml. Transferrin saturation and B2-microglobulin levels were normal. Antinuclear antibody (ANA) titer was 1:40 and rheumatoid factor was negative. As her pancytopenia deteriorated (WBC 3.9 K/μl, hemoglobin 8.8 g/dl, platelets 44 K/μl), she was started on steroids for a possible diagnosis of adult onset Still's disease (AOSD) with hemophagocytic syndrome. Bone marrow biopsy was done and was normocellular with trilineage hematopoiesis without any hemophagocytosis.

**Figure 1:**
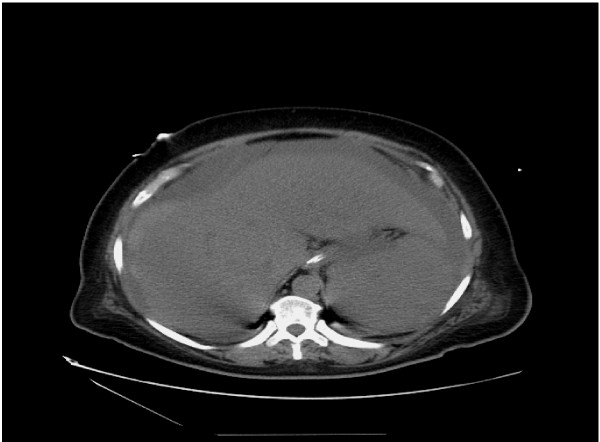
**Computed tomography scan of the abdomen without contrast after the liver biopsy showed acute hemorrhage (arrows).** The unenhanced liver is normal in size and attenuation.

**Figure 2:**
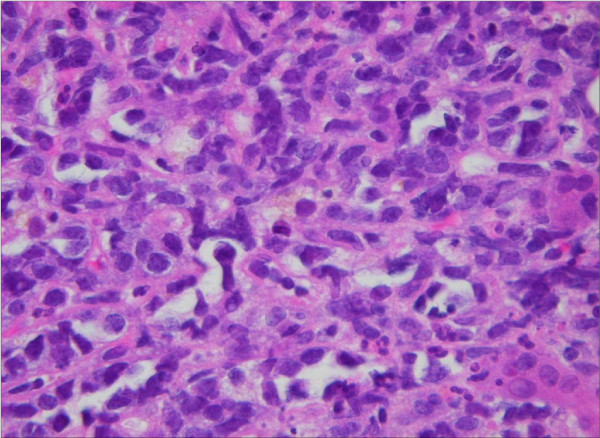
**Liver: Atypical large lymphoid infiltrates with distortion of hepatic parenchyma.** These cells were CD20+ confirming the B cell lineage.

After an initial improvement with steroids, liver function rapidly declined (Table 1). Hepatitis A, B, C, EBV, CMV, HIV as well as antismooth muscle and antimitochondrial serologies were negative. Abdominal ultrasound did not show any splenic, portal or hepatic vein thrombosis. Liver biopsy was delayed for almost a week because of the patient's persistent coagulopathy and arrangements were being made to transfer her to a liver transplant center. A percutaneous computed tomography (CT) guided liver biopsy was performed. Her clinical course was complicated by intra-abdominal hemorrhage (Figure 1) with shock, respiratory failure, hepatic encephalopathy, lactic acidosis and acute renal failure requiring temporary dialysis. Biopsy revealed diffuse large B cell lymphoma (Figure 2).

**Table 1:** Liver function tests

	Hospital day								
	
	1	5	6	9*	11	15	17	18	19
TBil	0.6	0.9	1.2	2.3	1.4	1.6	3.4	3.8	5.1
DBil	0.2	0.4	0.3	1.6	0.9	0.9	2.1	2.8	3.8
AST	67	265	326	771	467	384	812	1585	1868
ALT	28	80	83	234	217	209	435	522	538
ALP	57	214	204	307	252	247	416	386	364
Albumin	2.9	3.1	2.6	2.7	2.5	2.6	2.4	2.6	2.6
PT				16.6	17.6	17.2	20.6	20.0	20.9
INR				1.5	1.7	1.7	2.3	2.2	2.4

TBil, total bilirubin (0.3–1.3.mg/dl); DBil, direct bilirubin (0.0–0.2 mg/dl); AST, aspartate aminotransferase (8–46 U/liter); ALT, alanine aminotransferase (8–50 U/liter); ALP, alkaline phosphatase (25–125 U/liter); PT, prothrombin time (12–14.secs); INR, International Normalized Ratio. *Addition of steroids.

Chemotherapy was started immediately. The patient received a total of 6 cycles of chemotherapy; each cycle was given every 21 days. Initially she received 2 cycles of cyclophosphamide (1.5 g/m^2^) and rituximab and this was because vincristine and adriamycin were contraindicated due to her multi-organ failure. Though she showed improvement, the chemotherapeutic regimen was not felt to be adequate. She was then administered 2 cycles of R-DHAP (cytarabine and cisplatin salvage regimen mostly used for relapsed or refractory lymphoma). As her organ function recovered, she received another 2 cycles of R-CHOP (rituximab, cyclophosphamide, adriamycin, vincristine and prednisone).

A year into the diagnosis, she is in remission. CT scan does not show any liver or spleen enlargement and a recent positron emission tomography (PET) scan was also negative for lymphoma. Her blood counts, including liver enzymes and creatinine, have normalized (WBC 5 K/μl, platelets 161 K/μl, hemoglobin 13 g/dl). With the initiation of chemotherapy, the patient's ferritin levels also rapidly declined. Her most recent ferritin level is 449 ng/ml (range 13–150 ng/ml).

## Discussion

PHL is defined as lymphoma either confined to the liver or with major liver involvement [1]. Median survival is 8 to 16 months and complete remission is low (< 20%). It is important to recognize that in rare circumstances, it can present with fulminant hepatic failure (FHF) and because of the ambiguous features and rapid progression, most cases are diagnosed on autopsy with an average survival of 10.7 days from diagnosis [2].

It is twice as common in men and the usual age at presentation is 50 years. Exact etiology is unknown although viruses such as hepatitis B, C and Epstein-Barr have been implicated. Signs and symptoms can mimic a variety of infectious and inflammatory disorders delaying the diagnosis. A preliminary diagnosis of AOSD was made in this patient because of weeks of unexplained fever, hyperferritinemia, hepatitis, splenomegaly and a possible reactive hemophagocytic syndrome [3,4]. AOSD is however a diagnosis of exclusion. There have been reports of FHF with AOSD especially with concomitant use of acetaminophen and ibuprofen, and therapy and treatment with steroids can prevent liver transplantation [5]. For our patient, addition of steroids temporarily masked the clinical picture.

Ferritin is an acute phase reactant that can be elevated in a variety of clinical conditions including liver diseases (hemochromatosis), HIV, sepsis, malignancies including leukemias and lymphomas, and hemophagocytic syndrome. In healthy patients, 50% to 80% of ferritin is glycosylated, a process that provides a shield against the proteolytic enzymes. In inflammatory diseases, there is saturation of glycosylation mechanisms causing the glycosylated fraction to drop to 20% to 50%. This occurrence is particularly common in AOSD. Hence, combination of fivefold and greater elevation of serum ferritin and a glycosylated fraction of less than 20% has a sensitivity of 43% but a specificity of 93% for a diagnosis of AOSD [6]. A glycosylated ferritin level was not measured in our patient. Since very high ferritin levels are frequently associated with AOSD and hemophagocytic syndrome (HPS), it can be a red herring.

As for PHL, the most frequent pathology is diffuse large B cell followed by small lymphocytic, T cell, follicular and marginal B cell lymphoma. Fever, anorexia and upper quadrant pain are some of the symptoms. Though our patient had no evidence of hepatomegaly on serial imaging, liver enlargement is present in almost 80% of patients and solitary or multiple discrete masses are commonly found on computerized axial tomography (CAT) scan, ultrasound or MRI. Diffuse infiltration is rare and more common in the Chinese population [7]. Blood counts are initially normal and pancytopenia should alert the clinician to consider hemophagocytosis. More frequently associated with T cell lymphomas, HPS has also been described in B cell lymphomas [8]. Hemophagocytosis in the biopsy (bone marrow, lymph nodes, liver or spleen) is a prerequisite for diagnosis. Liver enzymes, including LDH, are elevated in most cases of PHL and hypercalcemia may be present. Alfa fetoprotein and carcinoembryonic antigen markers are normal in all patients [9].

Liver biopsy remains the most valuable tool for diagnosis of PHL. If a discrete mass is not visible on imaging for percutaneous liver biopsy (PLB), the transjugular approach may be reasonable. A recent review indicates that transjugular liver biopsy can be used to obtain adequate tissue samples and major complications and mortality rates are similar to PLB [10]. Our patient had significant hemorrhage after the PLB, despite correction of the prothrombin time to less than 15 seconds.

According to the Ann Arbor staging system, involvement of the bone marrow, lung and liver constitute Stage IV disease. Our patient was classified as Stage IV, because of diffuse liver involvement, rather than the traditional Stage IE classification by Caccamo and colleagues [11]. In her status, she would be expected to have a remission rate of just over 50% and a 5-year survival rate of around 25%.

Chemotherapy is the main treatment modality although surgery and radiotherapy have also been used. Standard CHOP chemotherapy can be challenging with severe hepatic dysfunction, and substantial dose reduction may be required. It is important to recognize and identify the causes of acute liver failure that require specific treatment, such as lymphoma, Budd-Chiari syndrome, or ischemic hepatitis [12]. While there have been a few cases of successful liver transplantation in PHL [13], the role is controversial. Liver histology is not routinely recommended in FHF [14]. However, in certain selective cases, timely recognition by liver biopsy can decrease the need for referral to a transplant center since an important variable for predicting the need of transplantation is the principal cause.

## Conclusion

PHL can manifest as progressive hepatitis and acute hepatic failure. The presence of constitutional symptoms, hematological abnormalities and altered acute phase reactants can complicate diagnostic evaluation.

If the clinical picture is suspicious for PHL, a liver biopsy should be attempted as soon as there is evidence of hepatitis because rapid progression can cause FHF and refractory coagulopathy with bleeding complications. Furthermore, early detection and timely initiation of combination chemotherapy can improve survival [15].

## Competing interests

The authors declare that they have no competing interests.

## Authors' contributions

FSH completed most of the manuscript after evaluation of the case, compilation of the data and literature review. RES reviewed the manuscript and contributed to the literature review regarding hepatic failure and hepatic lymphoma. SSK contributed to the interpretation of the histopathological data, the oncologic aspect of hepatic lymphoma and its chemotherapy.

## Consent

Written informed consent was obtained from the patient for publication of this case report and accompanying images. A copy of the written consent is available for review by the Editor-in-Chief of this journal.
